# New-Onset Focal to Bilateral Tonic-Clonic Seizure Following COVID-19 Vaccination

**DOI:** 10.1155/2024/8808334

**Published:** 2024-10-16

**Authors:** Chun Seng Phua, Azman Ali Raymond, Shalini Bhaskar

**Affiliations:** ^1^Monash University, Department of Neurosciences, Wellington Road, Clayton 3800, Victoria, Australia; ^2^Faculty of Medicine, Universiti Teknologi MARA, JLN Hospital, Sungai Buloh 47000, Selangor, Malaysia

**Keywords:** COVID-19, epilepsy, focal seizure, seizure threshold, vaccination

## Abstract

An 18-year-old male presented with new-onset focal to bilateral tonic-clonic seizures 5 days after receiving the first dose of COVID-19 vaccine. 3 days later, an identical seizure occurred lasting 1 min, leading to an acute presentation to the hospital. In hospital, the patient was loaded with intravenous phenytoin and started on levetiracetam with no further seizure recurrence. CT venogram and scalp EEG were unremarkable. MRI brain revealed generalised atrophy with mild bilateral hippocampal atrophy. The patient was maintained on 500 mg levetiracetam twice daily and advised to proceed with subsequent doses of vaccination. Vaccinations have been associated with breakthrough seizures. In this case, COVID-19 vaccination possibly unmasked an underlying predisposition for epilepsy.

## 1. Introduction

Since the COVID-19 pandemic started in year 2020, vaccinations against the COVID-19 virus were rapidly developed. While the novel vaccines were swiftly administered to the world's population, concern regarding vaccine safety and welfare naturally emerged, particularly in patients with comorbidities such as epilepsy. In patients with epilepsy, the concern was over seizure aggravation postvaccination. In this case report, we present a case of a healthy male with new-onset seizures following COVID-19 vaccination.

## 2. Case

We describe a case of a healthy 18-year-old male who developed new-onset focal to bilateral tonic-clonic seizures 5 days after receiving the first dose of COVID-19 vaccine. The focal seizures started with tonic posturing of the right hand which spread to the neck, followed by generalised tonic clonic seizure lasting 1 min. A second attack with identical semiology occurred 3 days later with involvement of the right hand at onset, which evolved to bilateral tonic-clonic seizures leading to an acute hospital admission day three postvaccination.

**Figure 1:**
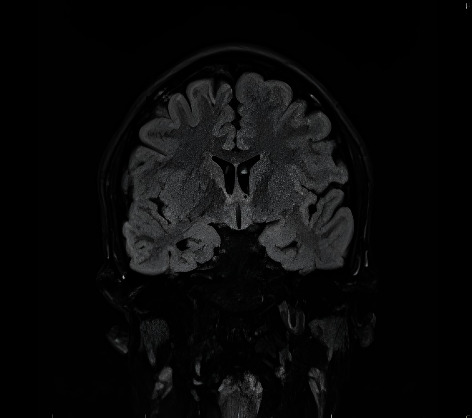
MRI brain coronal view showing generalised atrophy including mild bilateral hippocampal atrophy with no evidence of sclerosis.

Given the new-onset seizures, an initial blood glucose level was performed to rule out hypoglycaemia, which returned normal at 5.6 mmol/L. A venous blood gas showed a mildly elevated lactate of 4.1 mmol/L. Serological tests revealed a haemoglobin level of 134 g/L, total white cell count of 9.8 × 10^9^/L, platelet count of 402 × 10^9^/L, C reactive protein of 10 mg/L, normal coagulation profile and renal function tests. Rapid PCR test ruled out COVID-19 infection as the cause of seizures. An urgent CT brain did not reveal any abnormalities, and CT venogram was unremarkable. An MRI brain with contrast (epilepsy protocol) revealed generalised brain atrophy including mild bilateral hippocampal atrophy with no evidence of sclerosis (Figure 1). Routine scalp EEG done 24 h post event was unremarkable. This was not unexpected, as the sensitivity of routine scalp EEG is low [1]. There was no fever and no aura associated with the seizures. There were no remarkable events at birth, and all growth milestones were achieved appropriately. Patient had no mental health issues, and was performing well in school. The patient did not have any pre-existing conditions before receiving the vaccine. There was no history of epilepsy during childhood and no family history of epilepsy as corroborated by his parents.

The patient was loaded with intravenous phenytoin in the emergency department. He remained in a state of postictal confusion and drowsiness, where he was disoriented and responding inappropriately to questions before returning to his usual self an hour later. He was started on oral levetiracetam 500 mg twice daily with no further in-hospital events.

The patient was discharged one day after admission on levetiracetam. Given the possibility of seizure recurrence with subsequent vaccinations, patient was advised to proceed with the second dose of COVID-19 vaccination in 3 months' time while strictly adhering to the antiseizure medication.

## 3. Discussion

The possibility of infectious encephalitis was considered especially given the repeated events; however, given the normal infectious markers on blood test and quick recovery to baseline, infection was thought to be unlikely and lumbar puncture was not attempted. Autoimmune encephalitis was also considered; however, without an infective prodrome or any neuropsychiatric manifestations, this was unlikely. Furthermore, EEG did not show any encephalopathic changes. Despite the young age, acute stroke was not dismissed as a potential cause of seizures. CT venogram ruled out venous sinus thrombosis and MRI brain (epilepsy protocol) which included diffusion weighted imaging sequence ruled out stroke.

Vaccinations in general are a known trigger for seizures especially in the paediatric population [2]. Furthermore, vaccinations may unmask an underlying predisposition for epilepsy. During long-term follow-up, 65% of children who developed new-onset seizures after vaccination were found to have an underlying genetic or structural cause [3]. With regards to COVID-19 vaccination, seizures postvaccination has been reported infrequently in patients with underlying epilepsy. In one study involving 530 patients with epilepsy, 13 patients reported a seizure exacerbation following their first vaccination. Six of these patients had a further exacerbation of seizures with their second vaccination. An additional four patients reported increased seizures only with the second vaccine dose [4]. In another study of 54 patients with underlying epilepsy, one patient experienced increased seizure frequency a day after the first dose of COVID-19 vaccination, and one patient reported the occurrence of a new seizure type [5].

In this case report, the patient developed seizures with onset after vaccination, therefore, vaccination could have triggered an underlying predisposition to develop epilepsy. While the patient's brain atrophy is nonspecific, it may herald a predisposition for epilepsy. Studies have shown that most patients who developed seizures postvaccination had underlying genetic or structural defects such that the vaccine triggers, rather than causes seizures [6]. Furthermore, COVID-19 vaccination–induced seizures tend to occur more frequently in children [7]. The reduced seizure threshold could be through the release of inflammatory cytokines or fever induced because of immunization. Adjuvants and preservatives in the vaccine could potentially be triggering factors as well. Sleep disruption post–COVID-19 vaccination has been reported to lower seizure threshold [8]. In this case, the possibility of seizure recurrence is arguably high enough to justify the initiation of antiseizure medication, especially since the second and third doses of COVID-19 vaccination still need to be administered.

In conclusion, the International League Against Epilepsy strongly recommends COVID-19 vaccination for patients with epilepsy [9]. In patients with epilepsy, strict adherence to antiseizure medications during vaccination may help avoid seizure recurrence. In patients with no known epilepsy, vaccination may trigger the first onset of seizure, prompting further investigations for possible predilections for epilepsy.

## Data Availability

The authors have nothing to report.
